# Supplementary material for: A Likelihood Approach for Real-Time Calibration of Stochastic Compartmental Epidemic Models
Source: PLoS Comput Biol. 2017 Jan 17;13(1):e1005257. doi: 10.1371/journal.pcbi.1005257 (PMC5240920; doi:10.1371/journal.pcbi.1005257)
Supplement: S1 File — (TAR.GZ) [file pcbi.1005257.s014.tar.gz › HSPH_Online-SI-Revision/Additions/PF09/n100-extreme-SEITR/n100-extreme-SEITR_PF2_table8.pdf]

1	newly 3 weeks spec	108.	2015.3	{1963., 2075.}	1766.02	1.27082	newly 3 weeks cumul	716.	7387.69	{7290., 7468.}	931.8	1.01358	newly 5 weeks spec	52.	1354.9	{1305., 1403.}	2505.58	1.41572	newly 5 weeks cumul	854.	10 404.8	{10 320., 10 488.}	1118.36	1.08577	newly 43 weeks cumul	939.	16 048.9	{15 868., 16 203.}	1609.15	1.23276
2	newly 3 weeks spec	102.	1477.64	{1435., 1519.}	1348.67	1.16087	newly 3 weeks cumul	593.	6039.59	{5975., 6095.}	918.481	1.00794	newly 5 weeks spec	36.	834.12	{799., 870.}	2217.	1.36469	newly 5 weeks cumul	673.	7981.63	{7938., 8028.}	1085.98	1.07407	newly 50 weeks cumul	712.	10 453.	{10 390., 10 504.}	1368.12	1.16676
3	newly 3 weeks spec	169.	1605.42	{1561., 1651.}	849.953	0.977591	newly 3 weeks cumul	920.	6528.83	{6464., 6588.}	609.655	0.851034	newly 5 weeks spec	52.	909.22	{869., 952.}	1648.5	1.24237	newly 5 weeks cumul	1072.	8646.71	{8590., 8709.}	706.596	0.90665	newly 52 weeks cumul	1137.	11 349.	{11 281., 11 423.}	898.154	0.999193
4	newly 3 weeks spec	137.	1240.68	{1199., 1284.}	805.606	0.956774	newly 3 weeks cumul	790.	5085.45	{5027., 5147.}	543.728	0.808684	newly 5 weeks spec	41.	697.36	{657., 723.}	1600.88	1.23039	newly 5 weeks cumul	909.	6721.25	{6673., 6780.}	639.411	0.868878	newly 50 weeks cumul	985.	8781.89	{8712., 8848.}	791.562	0.950145
5	newly 3 weeks spec	34.	1653.25	{1598., 1704.}	4762.5	1.68674	newly 3 weeks cumul	171.	6723.41	{6665., 6788.}	3831.82	1.59458	newly 5 weeks spec	11.	934.84	{892., 976.}	8398.55	1.92911	newly 5 weeks cumul	195.	8895.13	{8845., 8955.}	4461.61	1.65911	newly 52 weeks cumul	201.	11 674.5	{11 603., 11 753.}	5708.19	1.76404
6	newly 3 weeks spec	5.	1382.53	{1336., 1419.}	27 550.6	2.44157	newly 3 weeks cumul	60.	5669.68	{5608., 5733.}	9349.47	1.97539	newly 5 weeks spec	2.	781.8	{746., 816.}	38 990.	2.59179	newly 5 weeks cumul	63.	7488.47	{7430., 7547.}	11 786.5	2.07505	newly 51 weeks cumul	64.	9793.8	{9723., 9852.}	15 202.8	2.18477
7	newly 3 weeks spec	469.	1999.65	{1922., 2055.}	326.365	0.629659	newly 3 weeks cumul	2142.	7406.68	{7326., 7487.}	245.783	0.53879	newly 5 weeks spec	221.	1333.8	{1275., 1385.}	503.529	0.780499	newly 5 weeks cumul	2717.	10 378.7	{10 296., 10 466.}	281.992	0.582045	newly 43 weeks cumul	3226.	15 835.	{15 677., 15 990.}	390.856	0.69094
8	newly 3 weeks spec	225.	1364.28	{1320., 1404.}	506.347	0.782574	newly 3 weeks cumul	1088.	5578.42	{5509., 5640.}	412.722	0.709865	newly 5 weeks spec	80.	771.47	{735., 805.}	864.338	0.983944	newly 5 weeks cumul	1321.	7375.96	{7316., 7431.}	458.362	0.746908	newly 50 weeks cumul	1472.	9664.74	{9599., 9725.}	556.572	0.817277
9	newly 3 weeks spec	19.	2008.69	{1944., 2063.}	10 472.1	2.02405	newly 3 weeks cumul	167.	8131.41	{8071., 8201.}	4769.11	1.68744	newly 5 weeks spec	5.	1137.77	{1092., 1177.}	22 655.4	2.35689	newly 5 weeks cumul	179.	10 789.9	{10 722., 10 851.}	5927.85	1.78016	newly 52 weeks cumul	188.	14 215.3	{14 137., 14 282.}	7461.32	1.87859
10	newly 3 weeks spec	613.	1434.56	{1387., 1490.}	134.023	0.369101	newly 3 weeks cumul	2661.	5748.06	{5660., 5818.}	116.011	0.334449	newly 5 weeks spec	260.	836.73	{802., 878.}	221.819	0.507312	newly 5 weeks cumul	3324.	7676.45	{7542., 7749.}	130.94	0.363478	newly 48 weeks cumul	3949.	10 298.6	{10 081., 10 408.}	160.789	0.416258
11	newly 3 weeks spec	448.	2981.29	{2916., 3045.}	565.467	0.823063	newly 3 weeks cumul	2137.	10 985.7	{10 897., 11 070.}	414.071	0.711015	newly 5 weeks spec	237.	1954.57	{1896., 2004.}	724.713	0.916192	newly 5 weeks cumul	2689.	15 367.9	{15 278., 15 459.}	471.509	0.757018	newly 47 weeks cumul	3221.	22 898.	{22 679., 23 087.}	610.896	0.851796
12	newly 3 weeks spec	209.	1336.6	{1292., 1380.}	539.522	0.805727	newly 3 weeks cumul	1086.	5399.07	{5343., 5454.}	397.152	0.696474	newly 5 weeks spec	68.	769.69	{739., 803.}	1031.9	1.05357	newly 5 weeks cumul	1294.	7192.69	{7139., 7245.}	455.849	0.74495	newly 48 weeks cumul	1442.	9562.98	{9479., 9627.}	563.175	0.82162
13	newly 3 weeks spec	300.	1697.48	{1646., 1748.}	465.827	0.752568	newly 3 weeks cumul	1508.	6770.19	{6700., 6840.}	348.952	0.652186	newly 5 weeks spec	131.	996.02	{947., 1035.}	660.321	0.880747	newly 5 weeks cumul	1818.	9060.42	{8989., 9127.}	398.373	0.697547	newly 49 weeks cumul	2109.	12 172.6	{12 090., 12 247.}	477.175	0.761302
14	newly 3 weeks spec	681.	2731.36	{2659., 2798.}	301.081	0.603146	newly 3 weeks cumul	2981.	9521.98	{9444., 9612.}	219.422	0.504356	newly 5 weeks spec	331.	1979.44	{1916., 2042.}	498.018	0.776584	newly 5 weeks cumul	3718.	13 837.8	{13 710., 13 948.}	272.183	0.570748	newly 42 weeks cumul	4474.	23 434.6	{23 158., 23 685.}	423.795	0.719147
15	newly 3 weeks spec	238.	3205.48	{3107., 3290.}	1246.84	1.12923	newly 3 weeks cumul	1233.	10 866.5	{10 750., 10 994.}	781.305	0.945113	newly 5 weeks spec	100.	2428.87	{2366., 2506.}	2328.87	1.38528	newly 5 weeks cumul	1479.	16 114.2	{15 959., 16 245.}	989.53	1.03723	newly 43 weeks cumul	1667.	28 876.9	{28 504., 29 140.}	1632.27	1.2386
16	newly 3 weeks spec	233.	1723.79	{1674., 1769.}	639.824	0.869018	newly 3 weeks cumul	1269.	6681.34	{6587., 6763.}	426.504	0.72138	newly 5 weeks spec	91.	1048.96	{1001., 1099.}	1052.7	1.06145	newly 5 weeks cumul	1497.	9077.29	{8964., 9169.}	506.365	0.782717	newly 46 weeks cumul	1647.	12 605.3	{12 426., 12 762.}	665.351	0.883832
17	newly 3 weeks spec	1.	1641.51	{1598., 1689.}	164 051.	3.21514	newly 3 weeks cumul	62.	6704.54	{6640., 6767.}	10 713.8	2.03396	newly 5 weeks spec	0.1	932.72	{887., 973.}	932 620.	3.96949	newly 5 weeks cumul	64.	8876.62	{8800., 8947.}	13 769.7	2.14206	newly 52 weeks cumul	64.	11 619.5	{11 553., 11 676.}	18 055.5	2.259
18	newly 3 weeks spec	93.	1677.57	{1619., 1730.}	1703.84	1.25609	newly 3 weeks cumul	605.	6815.73	{6749., 6883.}	1026.57	1.05174	newly 5 weeks spec	53.	956.42	{908., 1000.}	1704.57	1.25611	newly 5 weeks cumul	722.	9038.68	{8963., 9102.}	1151.89	1.09756	newly 51 weeks cumul	795.	11 883.7	{11 823., 11 943.}	1394.81	1.17458
19	newly 3 weeks spec	465.	2759.17	{2689., 2832.}	493.37	0.773237	newly 3 weeks cumul	2284.	10 178.6	{10 092., 10 267.}	345.646	0.64898	newly 5 weeks spec	217.	1813.39	{1754., 1864.}	735.664	0.9219	newly 5 weeks cumul	2812.	14 227.	{14 147., 14 320.}	405.937	0.704092	newly 46 weeks cumul	3336.	21 271.	{21 089., 21 427.}	537.619	0.804553
20	newly 3 weeks spec	75.	1405.57	{1366., 1450.}	1774.09	1.27266	newly 3 weeks cumul	401.	5758.58	{5697., 5820.}	1336.05	1.15715	newly 5 weeks spec	14.	787.52	{751., 817.}	5525.14	1.74991	newly 5 weeks cumul	459.	7601.54	{7541., 7650.}	1556.11	1.21908	newly 51 weeks cumul	486.	9936.79	{9867., 9998.}	1944.61	1.3106
21	newly 3 weeks spec	38.	1716.22	{1662., 1770.}	4416.37	1.65466	newly 3 weeks cumul	270.	6974.11	{6909., 7037.}	2483.	1.41211	newly 5 weeks spec	12.	969.71	{928., 1009.}	7980.92	1.90725	newly 5 weeks cumul	303.	9234.2	{9182., 9292.}	2947.59	1.48395	newly 52 weeks cumul	316.	12 107.1	{12 040., 12 173.}	3731.34	1.58335
22	newly 3 weeks spec	162.	1138.79	{1101., 1174.}	602.957	0.846766	newly 3 weeks cumul	806.	4638.89	{4577., 4705.}	475.545	0.760055	newly 5 weeks spec	58.	645.59	{609., 677.}	1013.09	1.04614	newly 5 weeks cumul	951.	6139.04	{6068., 6198.}	545.535	0.809907	newly 48 weeks cumul	1042.	8067.84	{8000., 8124.}	674.265	0.888882
23	newly 3 weeks spec	22.	1037.09	{1000., 1067.}	4614.05	1.67324	newly 3 weeks cumul	142.	4233.28	{4180., 4285.}	2881.18	1.47437	newly 5 weeks spec	3.	578.94	{546., 614.}	19 198.	2.28512	newly 5 weeks cumul	156.	5586.01	{5533., 5637.}	3480.78	1.55397	newly 49 weeks cumul	161.	7310.35	{7260., 7373.}	4440.59	1.6571
24	newly 3 weeks spec	51.	1855.15	{1804., 1899.}	3537.55	1.56071	newly 3 weeks cumul	312.	7519.78	{7463., 7581.}	2310.19	1.38204	newly 5 weeks spec	15.	1048.52	{992., 1098.}	6890.13	1.8442	newly 5 weeks cumul	360.	9964.78	{9896., 10 031.}	2667.99	1.44216	newly 51 weeks cumul	369.	13 098.9	{13 028., 13 179.}	3449.84	1.5502
25	newly 3 weeks spec	160.	1937.68	{1871., 1998.}	1111.05	1.08303	newly 3 weeks cumul	871.	7555.24	{7480., 7629.}	767.421	0.938212	newly 5 weeks spec	60.	1154.91	{1117., 1201.}	1824.85	1.28423	newly 5 weeks cumul	1024.	10 209.5	{10 111., 10 307.}	897.017	0.998689	newly 47 weeks cumul	1086.	13 952.5	{13 806., 14 127.}	1184.76	1.1088
26	newly 3 weeks spec	96.	1385.95	{1333., 1432.}	1343.7	1.1593	newly 3 weeks cumul	682.	5440.97	{5379., 5503.}	697.796	0.901877	newly 5 weeks spec	33.	829.79	{787., 866.}	2414.52	1.40017	newly 5 weeks cumul	784.	7343.01	{7251., 7411.}	836.608	0.971544	newly 45 weeks cumul	878.	10 082.4	{9942., 10 228.}	1048.33	1.06004
27	newly 3 weeks spec	572.	1461.3	{1416., 1514.}	155.472	0.407189	newly 3 weeks cumul	2438.	5751.58	{5675., 5826.}	135.914	0.372726	newly 5 weeks spec	284.	861.34	{821., 904.}	203.289	0.481555	newly 5 weeks cumul	3101.	7737.54	{7644., 7828.}	149.518	0.397076	newly 45 weeks cumul	3756.	10 539.	{10 366., 10 711.}	180.592	0.448037
28	newly 3 weeks spec	8.	1483.52	{1440., 1523.}	18 444.	2.26811	newly 3 weeks cumul	54.	6049.6	{5985., 6117.}	11 103.	2.04932	newly 5 weeks spec	1.	838.03	{798., 876.}	83 703.	2.923	newly 5 weeks cumul	61.	7999.52	{7951., 8049.}	13 014.	2.11773	newly 52 weeks cumul	63.	10 490.3	{10 430., 10 548.}	16 551.3	2.22144
29	newly 3 weeks spec	378.	1392.96	{1348., 1437.}	268.508	0.566318	newly 3 weeks cumul	1806.	5643.39	{5577., 5707.}	212.48	0.494806	newly 5 weeks spec	145.	786.51	{746., 824.}	442.421	0.734015	newly 5 weeks cumul	2196.	7475.84	{7415., 7523.}	240.43	0.53202	newly 49 weeks cumul	2473.	9843.95	{9771., 9925.}	298.057	0.599938
30	newly 3 weeks spec	146.	1857.05	{1804., 1911.}	1171.95	1.10436	newly 3 weeks cumul	831.	7547.07	{7484., 7622.}	808.191	0.958166	newly 5 weeks spec	47.	1044.26	{992., 1092.}	2121.83	1.34645	newly 5 weeks cumul	975.	9995.22	{9925., 10 056.}	925.151	1.01078	newly 52 weeks cumul	1051.	13 108.1	{13 021., 13 189.}	1147.2	1.09593
31	newly 3 weeks spec	292.	1284.48	{1243., 1326.}	339.89	0.643203	newly 3 weeks cumul	1423.	4935.91	{4865., 4994.}	246.866	0.54014	newly 5 weeks spec	119.	801.48	{766., 837.}	573.513	0.828095	newly 5 weeks cumul	1719.	6753.89	{6694., 6816.}	292.896	0.594268	newly 42 weeks cumul	1955.	9674.44	{9580., 9785.}	394.856	0.694463
32	newly 3 weeks spec	532.	1074.13	{1036., 1111.}	101.904	0.30499	newly 3 weeks cumul	2380.	4374.	{4317., 4425.}	83.7815	0.264281	newly 5 weeks spec	254.	601.33	{575., 627.}	136.744	0.373978	newly 5 weeks cumul	3005.	5780.71	{5724., 5838.}	92.3697	0.284123	newly 48 weeks cumul	3596.	7581.31	{7511., 7647.}	110.826	0.323912
33	newly 3 weeks spec	65.	1294.35	{1254., 1339.}	1891.31	1.299	newly 3 weeks cumul	390.	5315.62	{5263., 5366.}	1262.98	1.13448	newly 5 weeks spec	28.	727.09	{693., 759.}	2496.75	1.41416	newly 5 weeks cumul	468.	7015.37	{6966., 7065.}	1399.01	1.1758	newly 52 weeks cumul	508.	9142.67	{9094., 9200.}	1699.74	1.2552
34	newly 3 weeks spec	107.	995.88	{956., 1034.}	830.729	0.968641	newly 3 weeks cumul	548.	4061.56	{4008., 4113.}	641.161	0.869892	newly 5 weeks spec	35.	558.45	{531., 591.}	1495.57	1.20256	newly 5 weeks cumul	648.	5365.76	{5315., 5413.}	728.049	0.918044	newly 48 weeks cumul	692.	7035.59	{6976., 7092.}	916.704	1.00719
35	newly 3 weeks spec	10.	1671.39	{1619., 1716.}	16 613.9	2.22295	newly 3 weeks cumul	81.	6821.95	{6742., 6884.}	8322.16	1.92541	newly 5 weeks spec	0.1	953.5	{911., 991.}	953 400.	3.97909	newly 5 weeks cumul	82.	9038.33	{8983., 9101.}	10 922.4	2.04227	newly 52 weeks cumul	82.	11 860.9	{11 773., 11 939.}	14 364.6	2.1603
36	newly 3 weeks spec	587.	1481.93	{1434., 1530.}	152.458	0.402067	newly 3 weeks cumul	2638.	6032.42	{5966., 6094.}	128.674	0.3592	newly 5 weeks spec	275.	848.77	{808., 887.}	208.644	0.489195	newly 5 weeks cumul	3306.	8002.84	{7946., 8058.}	142.07	0.383931	newly 48 weeks cumul	3908.	10 559.3	{10 478., 10 645.}	170.196	0.431671
37	newly 3 weeks spec	138.	2154.07	{2099., 2208.}	1460.92	1.19329	newly 3 weeks cumul	721.	8571.89	{8495., 8648.}	1088.89	1.07513	newly 5 weeks spec	53.	1250.32	{1206., 1295.}	2259.09	1.37258	newly 5 weeks cumul	854.	11 463.6	{11 402., 11 523.}	1242.34	1.12786	newly 49 weeks cumul	922.	15 352.9	{15 259., 15 434.}	1565.17	1.22145
38	newly 3 weeks spec	103.	1575.32	{1499., 1646.}	1429.44	1.18427	newly 3 weeks cumul	598.	6188.77	{6046., 6348.}	934.911	1.01483	newly 5 weeks spec	29.	932.56	{885., 987.}	3115.72	1.50687	newly 5 weeks cumul	693.	8332.32	{8140., 8553.}	1102.35	1.07995	newly 46 weeks cumul	752.	11 356.9	{10 983., 11 745.}	1410.22	1.17888
39	newly 3 weeks spec	188.	1335.03	{1291., 1375.}	610.122	0.851211	newly 3 weeks cumul	946.	5371.28	{5309., 5434.}	467.789	0.754173	newly 5 weeks spec	61.	769.62	{730., 809.}	1161.67	1.10062	newly 5 weeks cumul	1112.	7157.76	{7102., 7214.}	543.683	0.808664	newly 48 weeks cumul	1224.	9542.58	{9465., 9604.}	679.623	0.891876
40	newly 3 weeks spec	395.	1051.88	{1010., 1094.}	166.299	0.425171	newly 3 weeks cumul	1868.	4284.01	{4233., 4345.}	129.337	0.360451	newly 5 weeks spec	218.	598.04	{563., 627.}	174.33	0.437899	newly 5 weeks cumul	2391.	5669.68	{5617., 5719.}	137.126	0.374968	newly 49 weeks cumul	2892.	7457.61	{7399., 7525.}	157.87	0.411392
41	newly 3 weeks spec	12.	1891.64	{1833., 1947.}	15 663.7	2.19754	newly 3 weeks cumul	121.	7671.29	{7613., 7739.}	6239.91	1.80207	newly 5 weeks spec	2.	1076.94	{1035., 1116.}	53 747.	2.73097	newly 5 weeks cumul	135.	10 179.9	{10 111., 10 247.}	7440.64	1.8774	newly 52 weeks cumul	137.	13 405.4	{13 333., 13 466.}	9684.96	1.99056
42	newly 3 weeks spec	675.	1556.88	{1496., 1611.}	130.649	0.362794	newly 3 weeks cumul	2966.	5950.64	{5879., 6024.}	100.628	0.302374	newly 5 weeks spec	340.	991.89	{957., 1035.}	191.732	0.464798	newly 5 weeks cumul	3794.	8181.98	{8101., 8270.}	115.656	0.333748	newly 43 weeks cumul	4690.	11 818.4	{11 660., 11 960.}	151.992	0.401368
43	newly 3 weeks spec	545.	1336.87	{1284., 1381.}	145.297	0.389546	newly 3 weeks cumul	2315.	5388.77	{5316., 5447.}	132.776	0.366922	newly 5 weeks spec	245.	769.64	{735., 805.}	214.139	0.496851	newly 5 weeks cumul	2918.	7178.19	{7119., 7234.}	145.997	0.390922	newly 48 weeks cumul	3370.	9569.64	{9487., 9654.}	183.966	0.453254
44	newly 3 weeks spec	215.	1272.25	{1220., 1317.}	491.744	0.771973	newly 3 weeks cumul	1211.	5177.59	{5110., 5238.}	327.547	0.630964	newly 5 weeks spec	93.	714.19	{678., 742.}	667.946	0.884994	newly 5 weeks cumul	1475.	6847.47	{6789., 6896.}	364.235	0.666729	newly 48 weeks cumul	1690.	8992.51	{8933., 9059.}	432.101	0.725988
45	newly 3 weeks spec	201.	1342.42	{1295., 1385.}	567.871	0.824558	newly 3 weeks cumul	1070.	5486.06	{5419., 5543.}	412.716	0.709861	newly 5 weeks spec	90.	756.44	{723., 785.}	740.489	0.924275	newly 5 weeks cumul	1275.	7256.53	{7203., 7312.}	469.14	0.755211	newly 50 weeks cumul	1419.	9505.9	{9446., 9557.}	569.901	0.826006
46	newly 3 weeks spec	138.	1592.67	{1554., 1638.}	1054.11	1.06214	newly 3 weeks cumul	722.	6473.87	{6401., 6542.}	796.658	0.952611	newly 5 weeks spec	50.	897.61	{857., 937.}	1695.22	1.25383	newly 5 weeks cumul	861.	8571.82	{8513., 8637.}	895.566	0.998062	newly 52 weeks cumul	942.	11 262.	{11 187., 11 334.}	1095.54	1.07756
47	newly 3 weeks spec	224.	2097.29	{2043., 2167.}	836.29	0.97131	newly 3 weeks cumul	1089.	8126.99	{8031., 8206.}	646.28	0.872886	newly 5 weeks spec	77.	1266.47	{1214., 1320.}	1544.77	1.21587	newly 5 weeks cumul	1298.	11 029.5	{10 916., 11 151.}	749.733	0.929269	newly 47 weeks cumul	1446.	15 280.1	{15 047., 15 490.}	956.714	1.02393
48	newly 3 weeks spec	86.	1458.55	{1420., 1498.}	1595.99	1.22933	newly 3 weeks cumul	493.	5953.73	{5882., 6013.}	1107.65	1.08193	newly 5 weeks spec	18.	820.25	{785., 860.}	4456.94	1.6584	newly 5 weeks cumul	561.	7866.75	{7803., 7929.}	1302.27	1.14683	newly 50 weeks cumul	581.	10 302.2	{10 248., 10 370.}	1673.18	1.24875
49	newly 3 weeks spec	117.	885.19	{852., 918.}	656.573	0.878665	newly 3 weeks cumul	638.	3615.2	{3568., 3656.}	466.646	0.75329	newly 5 weeks spec	40.	494.72	{467., 526.}	1136.8	1.09186	newly 5 weeks cumul	753.	4770.61	{4724., 4810.}	533.547	0.801768	newly 48 weeks cumul	828.	6240.74	{6186., 6290.}	653.713	0.877195
50	newly 3 weeks spec	261.	1111.12	{1069., 1155.}	325.716	0.628966	newly 3 weeks cumul	1311.	4548.61	{4489., 4615.}	246.957	0.540256	newly 5 weeks spec	104.	627.36	{594., 663.}	503.231	0.780105	newly 5 weeks cumul	1579.	6012.68	{5947., 6066.}	280.79	0.580673	newly 49 weeks cumul	1754.	7877.19	{7817., 7938.}	349.099	0.652334
